# Insecticide resistance intensity and efficacy of synergists with pyrethroids in *Anopheles gambiae* (Diptera: Culicidae) from Southern Togo

**DOI:** 10.1186/s12936-022-04377-2

**Published:** 2022-11-27

**Authors:** Yawo Apetogbo, Koffi M. Ahadji-Dabla, Dieudonné Diloma Soma, Adjovi D. Amoudji, Edoh Koffi, Kossivi I. Akagankou, Rabila Bamogo, Kelly Lionelle Ngaffo, Samina Maiga, Rachid T. Atcha‑Oubou, Ameyo M. Dorkenoo, Lucrecia Vizcaino, Audrey Lenhart, Abdoulaye Diabaté, Roch Kounbobr Dabiré, Guillaume Koffivi Ketoh

**Affiliations:** 1 Laboratoire d’Ecologie et d’Ecotoxicologie, Faculté des Sciences, Université de Lomé, 01 B.P. 1515, Lomé 01, Togo; 2 Institut de Recherche en Sciences de la Santé/Centre Muraz, 01 B.P. 545 , Bobo‑Dioulasso 01, Burkina Faso; 3 Programme National de Lutte contre le Paludisme/Ministère de la Santé, 01 B.P. 518, Lomé 01, Togo; 4 Faculté des Sciences de la Santé, Université de Lomé, 01 B.P. 1515, Lomé 01, Togo; 5 Center for Global Health, Division of Parasitic Diseases and Malaria/Entomology Branch, Centers for Disease Control and Prevention (CDC), Atlanta, GA 30329 USA; 6 Centre d’Excellence Africaine d’Innovation biotechnologique pour l’Elimination des Maladies à Transmission Vectorielle (CEA-ITECH-MTV), Université Nazi Boni, 01 B.P. 545 , Bobo‑Dioulasso 01, Burkina Faso

**Keywords:** Malaria, *Anopheles gambiae*, Vector control, Intensity resistance, Togo

## Abstract

**Background:**

This study was designed to provide insecticide resistance data for decision-making in terms of resistance management plans in Togo.

**Methods:**

The susceptibility status of *Anopheles gambiae sensu lato* (*s.l.*) to insecticides used in public health was assessed using the WHO tube test protocol. Pyrethroid resistance intensity bioassays were performed following the CDC bottle test protocol. The activity of detoxification enzymes was tested using the synergists piperonyl butoxide, S.S.S-tributlyphosphorotrithioate and ethacrinic acid. Species-specific identification of *An. gambiae s.l.* and *kdr* mutation genotyping were performed using PCR techniques.

**Results:**

Local populations of *An. gambiae s.l.* showed full susceptibility to pirimiphos methyl at Lomé, Kovié, Anié, and Kpèlè Toutou. At Baguida, mortality was 90%, indicating possible resistance to pirimiphos methyl. Resistance was recorded to DDT, bendiocarb, and propoxur at all sites. A high intensity of pyrethroid resistance was recorded and the detoxification enzymes contributing to resistance were oxidases, esterases, and glutathione-s-transferases based on the synergist tests. *Anopheles gambiae sensu stricto (s.s.)* and *Anopheles coluzzii* were the main species identified. High *kdr* L1014F and low *kdr* L1014S allele frequencies were detected at all localities.

**Conclusion:**

This study suggests the need to reinforce current insecticide-based malaria control interventions (IRS and LLINs) with complementary tools.

## Background

The use of insecticides is an important component of malaria vector control programmes in Africa [1]. However, the emergence of resistance to the main classes of insecticides used in the treatment of bed nets and in indoor residual spraying (IRS) requires re-thinking the use of these tools and the management of resistance in vectors [2]. Resistance has already been reported in various West African countries including Benin, Burkina Faso, Mali [3–5] and particularly in Togo [6, 7]. Recent studies showed that the use of synergists and combination of insecticide formulationscan increase the susceptibility of malaria vectors in areas with high pyrethroid resistance [8, 9]. To support the sustainability of control strategies, it is essential to consider that resistance management should be systematically integrated into any vector control policy [2]. The implementation of resistance management plans should be supported by the confirmation of resistance in any given country [10]. According to the World Health Organization (WHO) guidelines [10], resistance management involves the implementation of a three-step protocol including (1) assessment of insecticide susceptibility status of vectors, (2) characterization of resistance intensity, and (3) assessment of physiological resistance mechanisms with a focus on the efficacy of the synergist piperonyl butoxide (PBO). In Togo, the first step (i.e., evaluation of the susceptibility status of malaria vectors to insecticides) is carried out every 2 to 3 years at the National Malaria Control Programme (NMCP) sentinel sites. The last two steps (i.e., intensity of resistance and efficacy of the synergists piperonyl butoxide (PBO), S,S,S-tributyl phosphorotrithioate (DEF), and ethacrynic acid (EA)), are not yet widely conducted.

The present study aimed to address these three aspects to provide the NMCP with reliable data for decision-making regarding resistance management in Togo.

## Methods

### Study area

**Fig. 1:**
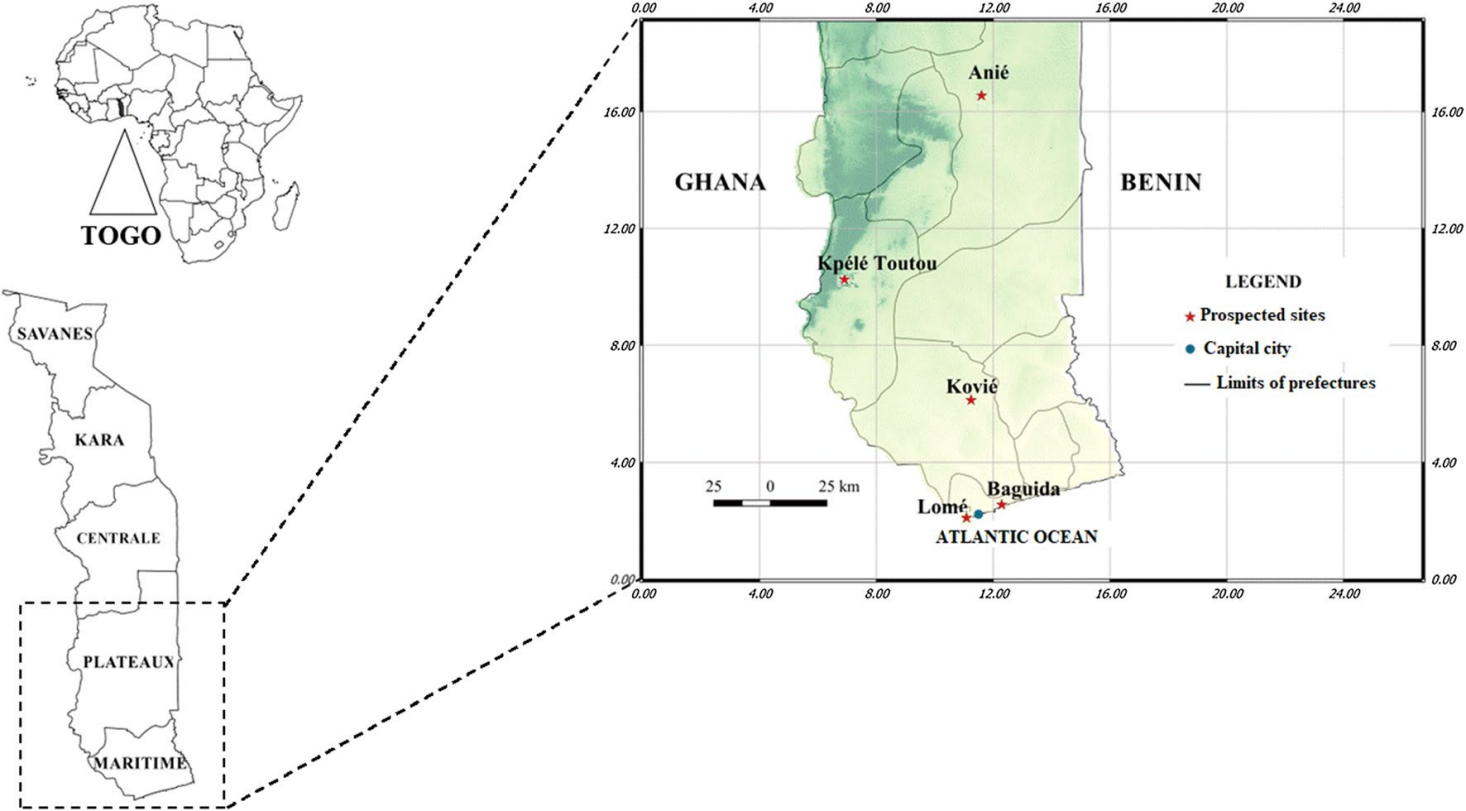
Map showing the localities surveyed

The present study was carried out at NMCP sentinel sites selected in three health regions in Southern Togo from June to September 2021 (Fig. 1). According to geographical (different health regions) and ecological characteristics (vector abundance, permanent larval breeding sites), five NMCP surveillance sites were selected for monitoring. The sites were Lomé, Baguida, Kovié, Anié, and Kpèlè Toutou (Table 1).

**Table 1:** Characteristics of monitoring sentinel sites

Sentinel sites	Description	Latitude	Longitude	Health region	Agricultural practices	Climate	Dry Seasons	Rainy Seasons
Lomé	Urban	31N0302166	UTM 0690055	Lomé	Capital of Togo	Sub-equatorial	April-July and October-November	December-March and August-September
Baguida	Peri-urban	31N0314169	UTM 0681627	Maritime	Vegetables	Sub-equatorial	April-July and October-November	December-March and August-September
Kovié	Peri-urban	31N0291756	UTM 0701889	Maritime	Rice	Sub-equatorial	April-July and October-November	December-March and August-September
Anié	Urban	31N0302493	UTM 0859340	Plateaux	Cotton and food crop	Sub-equatorial	April-July and October-November	December-March and August-September
Kpele Toutou	Rural	31N0250374	UTM 0785118	Plateaux	Rice	Sub-equatorial	April-July and October-November	December-March and August-September

### Mosquito collection and rearing

*Anopheles* larvae were collected from a minimum of 10 breeding sites located at least 150 m from each other at each sentinel site. Larvae were collected using methods described by Silver [11], pooled in 1.5 L plastic bottles and brought back to the Laboratory of Ecology and Ecotoxicology (LaEE)/Togo insectarium and reared under controlled conditions (temperature 25 ± 2 °C, Relative humidity 75 ± 2% and 12 L:12 D photoperiodicity) until adult emergence. Emerging adults were fed with 10% glucose solution.

### Insecticide susceptibility tests

Insecticide susceptibility tests were assessed on 3–5-day old females morphologically identified as *Anopheles gambiae sensu lato* (*s.l.*) using four classes of insecticides following the WHO standard protocol [10]. The tests were done with papers impregnated with three pyrethroids (deltamethrin 0.05%, permethrin 0.75%, and alphacypermethrin 0.05%), one organochlorine (DDT 4%), two carbamates (bendiocarb 0.1% and propoxur 0.1%), and one organophosphate (pirimiphos-methyl 0.25%). For each insecticide paper, four replicates of 20–25 unfed females were exposed for one hour. *Anopheles gambiae* “Kisumu” strain was used as a reference susceptible control strain in each bioassay. Mortality was recorded 24 h after exposure.

### CDC bottle intensity assays

Resistance intensity of *An. gambiae* to insecticides was performed using the CDC bottle bioassay protocol [12]. Three pyrethroids were tested at the following doses: deltamethrin (12.5 µg, 25 µg, 62.5 and 125 µg), permethrin (21.5 µg, 43 µg, 107.5 and 215 µg) and alphacypermethrin (12.5 µg, 25 µg, 62.5 and 125 µg). Approximately 20 unfed females aged 3 to 5 days old were introduced into a CDC bottle. The CDC bottle (250 ml) was previously impregnated with 1ml of the insecticide solution to be evaluated and dried under laboratory conditions for 24 h. Mosquitoes were exposed for 60 min. Mortality was recorded every 15 min for one hour. Four bottles were used for each concentration of insecticide. Control bottles were impregnated with ethanol.

### Synergist tests

Papers impregnated with the synergist piperonyl butoxide (PBO 4%) and bottles impregnated with S.S.S-tributlyphosphorotrithioate (DEF 125 µg) and ethacrynic acid (EA 80 µg) were used for the synergist tests. For synergist testing with PBO, mosquitoes were pre-exposed to PBO papers for one hour before being exposed to pyrethroids (deltamethrin, permethrin and alphacypermethrin) using the WHO tube testing protocol [10]. Mosquitoes were exposed to DEF synergist for one hour before exposure to organophosphates (malathion 50 µg and fenitrothion 50 µg) and carbamates (bendiocarb 12.5 µg) and EA for 1 hour before exposure to organochlorines (DDT 100 µg) using the CDC bottle testing protocol [12]. After bioassay testing, mosquitoes were preserved in Eppendorf tubes containing silica gel and stored at − 20 °C for molecular analyses.

### Molecular analysis

Molecular analysis was performed on a subsample of 50 females randomly selected per site at the IRSS laboratory / Burkina Faso. DNA was extracted from head-thorax of *An. gambiae* with 2% Cetyl Trimethyl Ammonium Bromide (2% CTAB). Members of the *An. gambiae* complex were identified using the PCR-SINE 200X (Short INterspersed Elements) technique of Santolamazza et al. [13].

Detection of the West (*kdr-w*) and East African *kdr* (*kdr-e*) mutations were performed following the protocols of Martinez-Torres et al. [14] and Ranson et al. [15], respectively.

### Data analysis

Data were entered using Microsoft Office Excel 2016 software. Allelic frequencies for each *kdr* mutation were compared between sites for each species using the “G-test” [16] performed in Genepop 4.7 and run with R software (version 4.0.3) [17].

Resistance intensity at 5 x and 10 x diagnostic doses was interpreted according to CDC criteria whereby mosquitoes were categorized as ‘dead’ if they were immobilized by the effect of the insecticide, and unable to stand or fly. The diagnostic time for all insecticides used was 30 min except for DDT which is 45 min [10]. Mortality rates were calculated and interpreted according to the following thresholds and criteria [10] :

- Mortality < 90%: Resistant.
- 90% ≤ mortality ≤ 97%: Probable Resistant.
- Mortality ≥ 98%: Susceptible.

## Results

### Susceptibility status of *An*. *gambiae* to insecticides

**Fig. 2:**
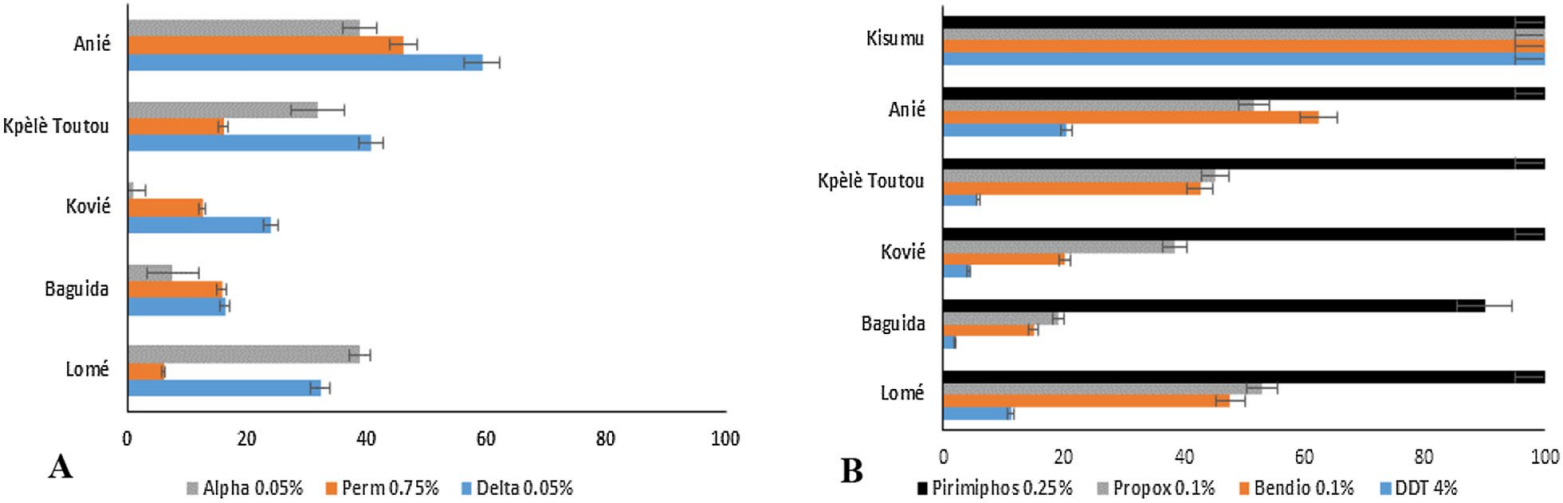
Susceptibility status of *An. gambiae s.l.* to pyrethroids (**A**) and to organophosphates, carbamates, and DDT (**B**). *Alpha* Alphacypermethrin, *Perm* Permethrin, *Delta* Deltamethrin, *Propox* Propoxur, *Bendio* Bendiocarb

Mortality rates of the Kisumu strain of *An. gambiae* was 100% with all insecticides tested (Fig. 2). Local populations of *An. gambiae* showed full susceptibility to pirimiphos methyl at four (4) localities (Lomé, Kovié, Anié and Kpèlè Toutou) with mortality rates of 100%. At Baguida, mortality was 90%, indicating possible resistance to pirimiphos methyl.

Susceptibility tests showed resistance to DDT (mortality ranging between 2% and 20.5%), bendiocarb (15–62.5%), propoxur (19.25–51.75%), deltamethrin (16.25–59.25%), permethrin (6–46%), and alphacypermethrin (1–38.75%) across all study sites (Fig. 2).

### Resistance intensity of *An*. *gambiaes*.*l*. to pyrethroids

Resistance intensity tests of local populations of *An. gambiae* exposed to pyrethroids (deltamethrin, permethrin and alphacypermethrin) showed mortality rates of less than 98% at the 5 x and 10 x diagnostic doses after 30 min. The highest mortality rates were 90.3% at Kpèlè Toutou (Table 2), 90.7% at Anié (Table 3) and 90.8% at Baguida (Table 4) for deltamethrin, permethrin and alphacypermethrin, respectively. These results reflect a high resistance intensity of *An. gambiae* to pyrethroids at all localities surveyed.

**Table 2:** Resistance intensity of *An. gambiae s.l.* 30 min after exposure to deltamethrin

Strain	Deltamethrin								Status
	1 × (12,5 µg)		2 × (25 µg)		5 × (62,5 µg)		10 × (125 µg)		
	n	% Mortality [CI]	n	% Mortality [CI]	n	% Mortality [CI]	n	% Mortality [CI]	
*An. gambiae* « Kisumu »	89	100	86	100	85	100	96	100	Low intensity
Lomé	88	45.64 [38.22–53.07]	88	80.64 [77.27–84]	91	87.03 [83.03–91.03]	85	91.78 [88.19–95.37]	High intensity
Baguida	79	79.7 [7316–86.23]	83	87.97 [84.04–91.91]	74	89.05 [86.80–91.3]	80	91.18 [86.86–95.49]	High intensity
Kovié	91	77.49 [67.07–87.91]	93	79.38 [72.1–86.66]	90	84.06 [65.48–102.6]	97	91.69 [90.38–93.01]	High intensity
Kpèlè Toutou	83	69.47 [58.86–80.08]	83	79.72 [75.48–83.95]	87	80.88 [70.82–90.95]	88	94.28 [90.63–97.93]	High intensity
Anié	83	75.41 [64.96–85.86]	92	89.15 [85.52–92.77]	83	89.22 [86.73–91.72]	90	91.23 [85.97–96.48]	High intensity

*n* Number of mosquitoes, *CI* Confidence Interval

**Table 3:** Resistance intensity of *An. gambiae s.l.* 30 min after exposure to permethrin

Strain	Permethrin								Status
	1 × (21,5 µg)		2 × (43 µg)		5 × (107,5 µg)		10 × (215 µg)		
	n	% Mortality [CI]	n	% Mortality [CI]	n	% Mortality [CI]	n	% Mortality [CI]	
*An. gambiae* « Kisumu »	89	100	68	100	79	100	81	100	Low intensity
Lomé	84	22.47 [14.49–30.44]	82	35.25 [28.75–41.74]	86	55.89 [48.71–63.07]	80	86.09 [81.03–91.16]	High intensity
Baguida	84	33.99 [21.48–46.5]	91	68.97 [41.98–95.96]	91	83.74 [79.47–88.02]	83	86.72 [82.81–90.63]	High intensity
Kovié	92	0	94	45.78 [39.62–51.95]	76	69.06 [57.38–80.74]	89	88.67 [84.37–92.97]	High intensity
Kpèlè Toutou	83	5.01 [0–11.6]	79	80.22 [67.44–92.99]	97	77.11 [65.79–88.43]	83	76.15 [63.86–88.44]	High intensity
Anié	82	19.51 [13.68–25.34]	83	42.36 [29.57–55.15]	81	85.62 [77.22–94.02]	76	90.68 [85.9–95.45]	High intensity

*n* Number of mosquitoes, *CI* Confidence Interval

**Table 4:** Resistance intensity of *An. gambiae s.l.* 30 min after exposure to alphacypermethrin

Sites	Alphacypermethrin								Status
	1 × (12,5 µg)		2 × (25 µg)		5 × (62,5 µg)		10 × (125 µg)		
	N	% Mortality [CI]	n	% Mortality [CI]	n	% Mortality [CI]	n	% Mortality [CI]	
*An. gambiae* « Kisumu »	95	100	98	100	95	100	98	100	Low intensity
Lomé	80	36.81 [25.83–47.79]	90	73.39 [70.18–76.59]	86	87.26 [84.23–90.28]	95	88.65 [85.69–91.61]	High intensity
Baguida	97	75 [67.32–82.68]	93	80.62 [77.22–84.02]	89	87.61 [83.98–91.23]	88	90.84 [89.48–92.2]	High intensity
Kovié	82	2.17 [0-6.92]	87	81.46 [74.15–88.78]	91	84.16 [79.08–89.24]	100	87.58 [76.27–98.89]	High intensity
Kpèlè Toutou	89	2.44 [0-6.92]	88	64.04 [53.56–74.51]	90	80.10 [74.03–86.18]	82	84.41 [80.54–88.29]	High intensity
Anié	74	70.73 [60.86–80.61]	72	74.41 [64.13–84.69]	75	86.64 [83.5-89.77]	78	84.56 [82.93–86.19]	High intensity

*n* Number of mosquitoes, *CI* Confidence Interval

### Efficacy of synergists in restoring susceptibility to insecticides

**Fig. 3:**
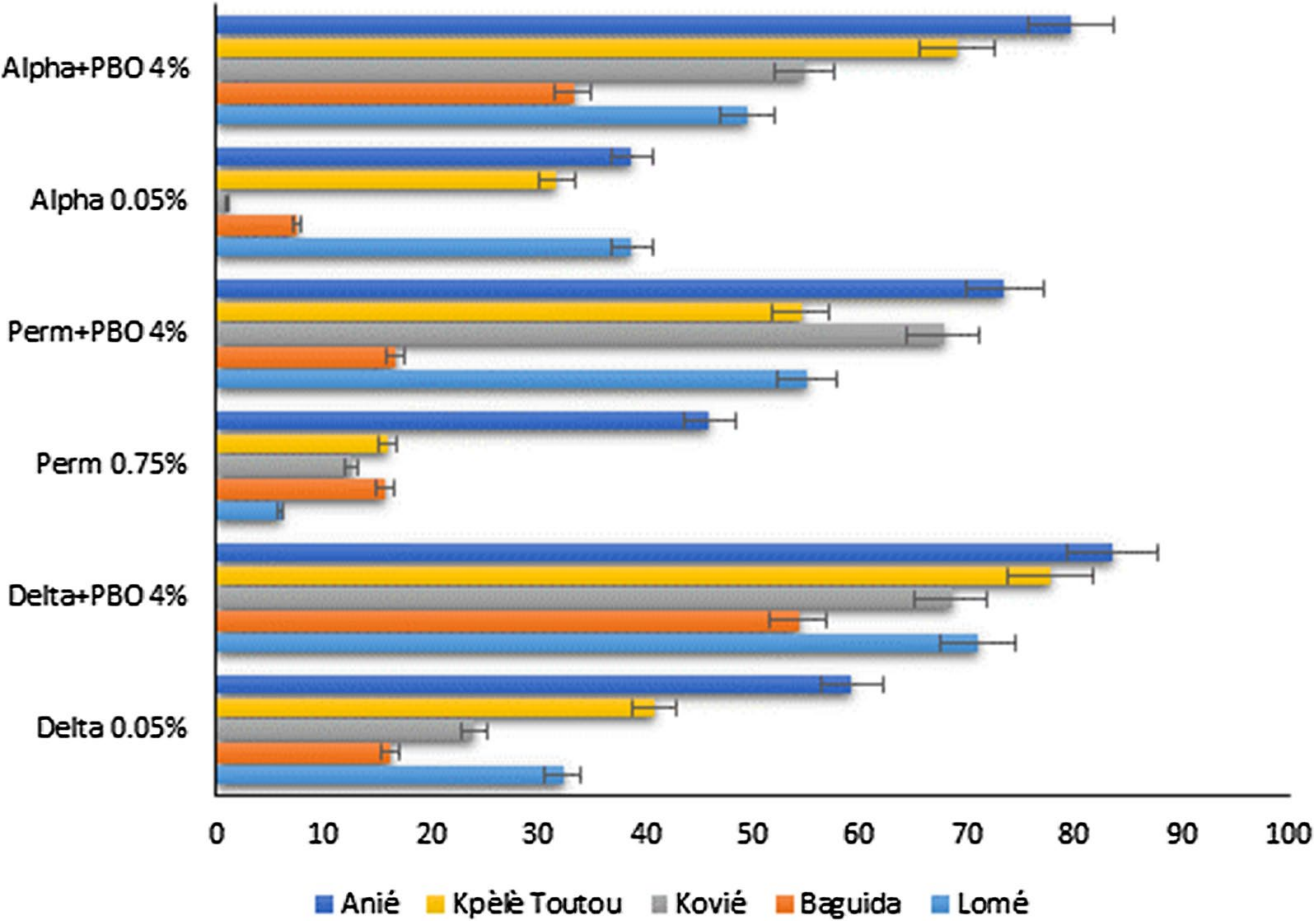
Susceptibility status of *Anopheles gambiae s.l.* to pyrethroids with pre-exposure to PBO *Delta* deltamethrin, *Perm* permethrin, *Alpha* alphacypermethrin, *PBO* Piperonyl Butoxide

Local populations of *An. gambiae* were resistant to deltamethrin, permethrin and alphacypermethrin with mortality rates ranging from 1 to 60% at all sites. The use of the synergist PBO partially restored the susceptibility of *An. gambiae* to pyrethroids with mortality rates ranging from 16.8 to 83.5% across all sites (Fig. 3).

**Fig. 4:**
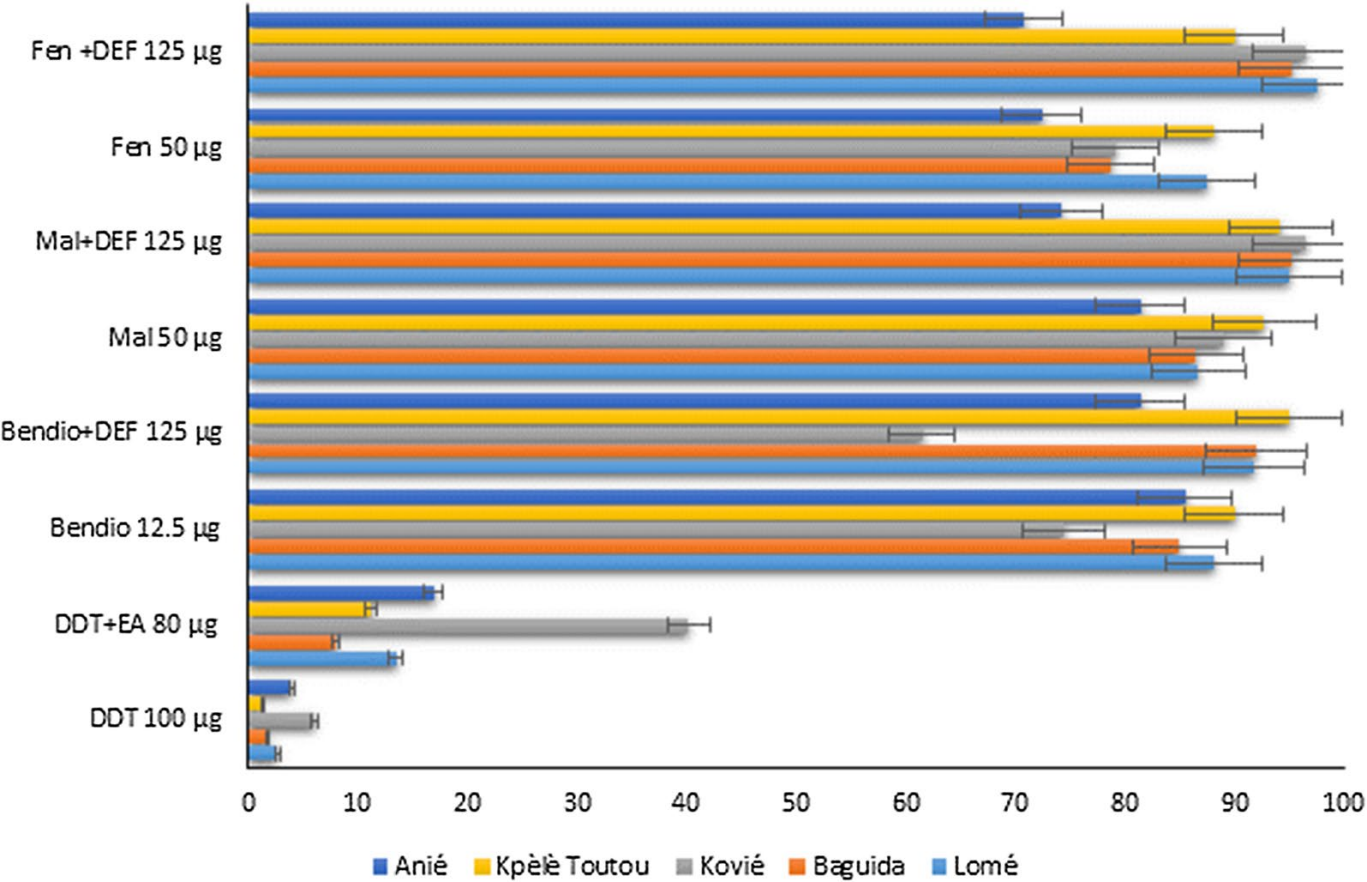
Susceptibility status of *An. gambiae s.l.* to DDT with pre-exposure to EA and to bendiocarb, malathion, and fenitrothion with pre-exposure to DEF *Bendio* bendiocarb, *Mal* malathion, *Fen* fenitrothion, *EA* ethacrinic acid, *DEF* S.S.S-tributlyphosphorotrithioate

Mortality rates were less than 98% for local populations of *An. gambiae* across all sites for DDT, fenitrothion, malathion and bendiocarb. Using the synergist EA, partial restoration of susceptibility was obtained to DDT at all sites with mortality ranging from 8 to 40.3%. The synergist DEF partially restored the susceptibility of *An. gambiae* to bendiocarb in Lomé, Baguida and Kpèlè Toutou (mortality: 91–95%), but not as much at Anié and Kovié where mortality rates were 81.5% and 61.5%, respectively (Fig. 4). The synergist DEF partially restored the susceptibility of *An. gambiae* to organophosphates (fenitrothion and malathion) at all sites except at Anié with mortalities ranging from 70.75 to 74.25%.

### Species composition of *An*. *gambiae* (*s*.*l*.)

**Fig. 5:**
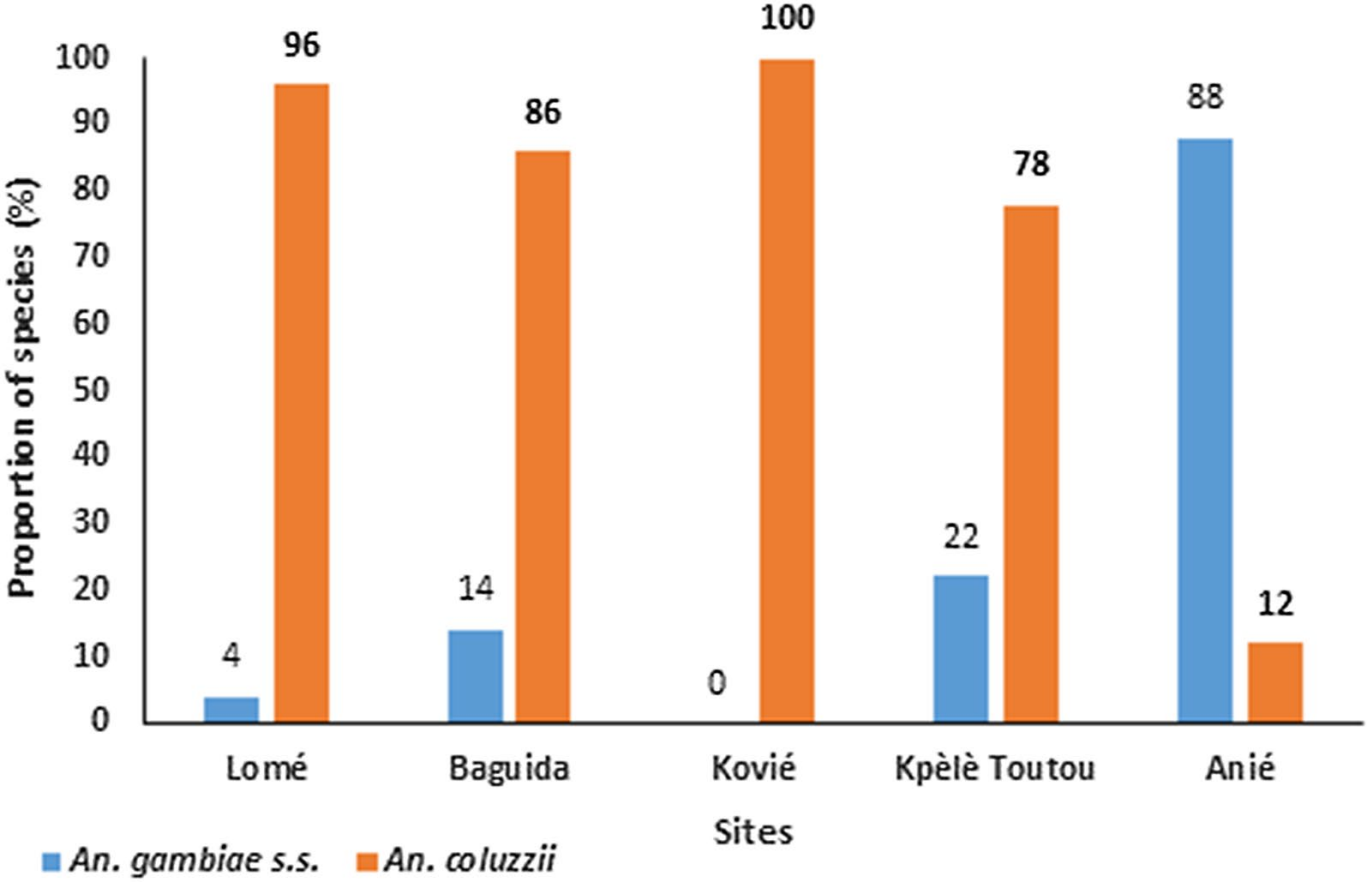
Distribution of the sibling species of *Anopheles gambiae s.l*

A total of 250 *An. gambiae (s.l.)* were analysed by PCR for species identification of the *An. gambiae* complex. The successfully identified species were *An. gambiae sensu stricto* (*s.s.*) (26.4%, n = 64) and *An. coluzzii* (73.6%, n = 178) in varying proportions depending on the locality (Fig. 5). Eight (8) mosquitoes from Kovié were PCR negative (i.e., undetermined species). *Anopheles coluzzii* was the predominant species in Lomé, Baguida, Kovié and Kpèlè Toutou with proportions varying from 78 to 100% (Fig. 5) whereas *An. gambiae s.s.* predominated in Anié with a proportion of 88%.

### *Kdr*-*west* (1014 F) and *kdr*-*east* (1014 S) allele frequencies

Molecular analyses detected the *kdr* L1014F mutation in *An. gambiae s.s.* and *An. coluzzii* (Table 5). The frequencies of this *kdr* mutation were relatively high (> 0.70) at all sites except Anié where a lower frequency of 0.50 was observed in *An. coluzzii*. In contrast, the *kdr* L1014S mutation was found only in *An. coluzzii* with low frequencies in Lomé (0.08), Kovié (0.02) and Kpèlè Toutou (0.01) (Table 5). When comparing the frequencies of the *kdr* L1014F mutation between the populations of *An. coluzzii* and *An. gambiae s.s.*, no statistically significant difference was detected between the different study sites (G exact test; p > 0.12). The low frequencies of the *kdr* L1014S mutation did not allow comparison between study sites and between species.

**Table 5:** Allelic frequencies of *kdr L1014F* and *L1014S* in the *An. gambiae* complex

Species	Sites	n	Genotypes			f(1014F)	Genotypes			f(1014S)
			SS	RS	RR		SS	RS	RR	
*An. gambiae s.s.*	Lomé	2	0	1	1	0.75	2	0	0	0
	Baguida	7	0	1	6	0.93	7	0	0	0
	Kovié	–	–	–	–	–	–	–	–	–
	Kpèlè Toutou	11	0	0	11	1	11	0	0	0
	Anié	44	0	2	42	0.98	44	0	0	0
*An. coluzzii*	Lomé	48	0	22	26	0.77	40	8	0	0.08
	Baguida	43	4	21	18	0.66	43	0	0	0
	Kovié	42	2	15	25	0.77	40	2	0	0.02
	Kpèlè Toutou	39	4	14	21	0.72	38	1	0	0.01
	Anié	6	2	2	2	0.5	6	0	0	0

*n* number of mosquitoes, *SS* homozygote susceptible, *RS* heterozygote, *RR* homozygote resistant, f(1014 F); kdr-west mutation frequency, f(1014 S); kdr-east mutation frequency, -; not determined

## Discussion

This study was conducted to update the insecticide resistance status of *An. gambiae s.l.* in key populations in Togo. The susceptibility tests showed that all local strains of *An. gambiae s.l.* were resistant to the insecticides tested except to pirimiphos methyl, where susceptibility was detected except for the strain from Baguida which showed possible resistance. The widespread use of insecticide provides a selection pressure for resistance in malaria vectors across Africa. Elsewhere in West Africa, evidence of a relationship between insecticide use in agriculture and the emergence of insecticide resistance in malaria vectors has been reported by several authors [18, 19]. These authors showed that mortality to deltamethrin was particularly low in market gardening areas [19]. In this study, the resistance was also high at Baguida, also a market gardening area. In the same area, there was probable resistance to pirimiphos methyl; this could be due to the introduction and regular use of organophosphates as an alternative to pyrethroids, the only WHO recommended class of insecticides used in market gardening. In addition, high levels of resistance to pyrethroids (deltamethrin, permethrin, and alphacypermethrin) were recorded in all localities.

Managing insecticide resistance remains the major challenge to achieving effective malaria vector control [2]. National and regional efforts through insecticide rotation and the introduction of new insecticide classes appear to be unsuccessful because of the intensity and progression of pyrethroid resistance [20]. Pyrethroids, DDT, bendiocarb, and propoxur induced low mortality rates at all localities tested, suggesting cross resistance and the presence of multiple resistance mechanisms. Resistance mechanisms, especially those involved in pyrethroid resistance, could have been selected by the large-scale use of long-lasting insecticide-treated nets (LLINs) throughout the country, as reported by Protopopoff et al. [21]. In urban areas, the use of spirals and aerosols could also contribute to insecticide resistance, as it is the case with market gardening practices [22]. Studies by Agboyi et al. [23] and Mondedji et al. [24] in Togo showed that farmers overuse synthetic pesticides for plant protection against pests. According to Agboyi et al. [23], while DDT and its derivatives are banned, they are still used by farmers in cotton and vegetable fields. Insecticide residues accumulate in the soil so that during the rainy season, breeding sites become contaminated. The exploitation of hydro-agricultural developments following the creation of agropoles could favour the introduction of new insecticide groups. In Ghana, Pwalia et al. [25] also reported a high intensity of resistance to multiple insecticides (deltamethrin, permethrin, DDT, bendiocarb, propoxur and pirimiphos methyl) using CDC bottle bioassays.

High frequencies of *kdr-west* alleles varying from 0.5 to 1 were detected. The *L1014S* allele (*kdr-east*) was detected at very low frequencies and was present in Lomé, Kovié and Kpèlè Toutou at proportions ranging from 0.01 to 0.08% in *An. coluzzii* only. Amoudji et al. [7] reported high *L1014F* allele frequencies (~ 0.9) at Baguida, Kovié and Kolokopé and low frequencies of *L1014S* allele (~ 0.02). These two alleles contribute to cross-resistance to organochlorines and pyrethroids. These results are consistent with reports by Diabaté et al. [26] and Yadouleton et al. [27] in Burkina Faso and Benin, respectively, which showed that *1014 F* allele frequency in *An. gambiae s.l.* is higher in cash crop areas usually subjected to insecticide treatments than in rural areas where farmers only grow food crops or products for local consumption. No individual *An. gambiae s.s.* was homozygous SS for the *kdr-west* genotype. However, the proportions of heterozygous RS and homozygous RR individuals were high, suggesting strong selection pressure.

In this study, three groups of detoxification enzymes were indirectly incriminated through the testing with PBO, DEF and EA. The use of the synergists PBO with pyrethroids, EA with DDT and DEF with organophosphates and carbamates, restored partially the susceptibility of the tested mosquito populations. This is an indication of the overproduction of oxidases, glutathione-s-transferases and esterases, respectively. Similar findings were reported by Namountougou et al. [28]. It should be noted that ethacrynic acid is a synergist for GSTs with peroxidase activity specifically. This may explain the partial susceptibility restoration observed.

The use of LLINs that contain PBO can be encouraged for resistance management based on our results. Hien et al. [9] and Ahadji-Dabla et al. [29] showed that PBO partially restored the susceptibility of *An. gambiae s.l.* to deltamethrin, alphacypermethrin and permethrin in Burkina Faso and Togo, respectively. Moreover, recent report by Ketoh et al. [8] showed the efficacy of PBO LLINs in an area with pyrethroid resistant vector populations. Similarly, DEF and EA could be recommended as adjuvants. A study by Aïzoun et al. [30] showed that PBO, DEF and EA partially restored the susceptibility of *An. gambiae s.l.* to deltamethrin, permethrin and DDT in southern Benin. According to these authors, a combination of PBO and DEF better restored the susceptibility than these synergists used individually.

Two species of the *An. gambiae* complex (*An. gambiae s.s*. and *An. coluzzii*) were identified in different proportions according to the locality. The predominant species were *An. coluzzii* in Lomé, Baguida, Kpèlè Toutou and Kovié and *An. gambiae s.s* in Anié. This situation could be the result of the adaptability to suitable environmental conditions. In addition, the predominance of *An. coluzzii* in the four localities mentioned above could also be explained by the presence of irrigated perimeters and permanent water bodies in rice and vegetable-growing areas. Recently, Toglo et al. [31] reported that *An. coluzzii* was the only species found in Kovié.

In Anié which is primarily a cotton-growing area, most of the breeding sites were temporary and were colonized by *An. gambiae s.s.* Similar findings were reported by Della Torre et al. [32] and Costantini et al. [33]. In Burkina Faso, Diabaté et al. [34] found that *An. coluzzii* was the primary species found in areas with permanent breeding sites, while *An. gambiae s.s.* was found in areas with temporary breeding sites. *Anopheles arabiensis*, classically considered a dryland or dry season species, was not detected in this study; however, it was previously identified at low proportions at Baguida, Kovié and Kolokopé by Amoudji et al. [7]. Unfavourable environmental conditions could affect the development of this species as reported by Duvallet et al. [35].

## Conclusion

This study revealed the existence of resistance to multiple key public health insecticides in the local populations of *An. gambiae s.l.* in southern Togo, except for pirimiphos methyl. High intensity pyrethroid resistance was observed at the study localities with probable involvement of detoxification enzymes (oxidases, esterases and glutathione-s-transferases). The *kdr* L1014F mutation was detected with variable but high allele frequencies (> 0.50) in the two sibling species of *An. gambiae s.s*. and *An. coluzzii* whereas the *kdr* L1014S mutation was present at very low frequencies and only in *An. coluzzii*. The synergists PBO and EA partially restored the susceptibility to pyrethroids and organochlorines, respectively, at all localities and DEF improved the susceptibility to carbamates and organophosphates at all localities except Anié. These data may help the NMCP of Togo in developing more effective strategies to control malaria vectors.

Further investigations are needed to understand the extent of resistance. It would be useful to (1) extend the study of resistance intensity to all the NMCP sentinel sites, (2) test the efficacy of newer public health insecticides such as chlorfenapyr (pyrrole) and clothianidin (neonicotinoid), (3) explore other resistance mechanisms (*ace-1* and *kdr* N1575Y) and (4) assess the impact of resistance on the efficiency of vector control tools to prevent malaria in Togo, especially LLINs and IRS.

## Data Availability

All data generated or analysed during this study are included in this manuscript.

## Abbreviations

CTAB: cetyl trimethyl ammonium bromide
DEF: S.S.S-tributlyphosphorotrithioate
DDT: dichlorodiphenyltrichloroethane
DNA: desoxyribonucleic acid
EA: ethacrynic acid
IRSS: Institut de Recherche en Sciences de la Santé
kdr: knock down resistance
LLIN: long-lasting insecticide-treated nets
NMCP: National Malaria Control Programme
PBO: Piperonyl butoxide
PCR: polymerase chain reaction
PNLP: Programme National de Lutte contre le Paludisme
(RR): resistant homozygous
SINE 200: Short INterspersed Elements
(SS): susceptible homozygous
WHO: World Health Organization.
